# Extended-spectrum β-lactamase- producing gram-negative bacterial infections in severely ill COVID-19 patients admitted in a national referral hospital, Kenya

**DOI:** 10.1186/s12941-023-00641-8

**Published:** 2023-10-14

**Authors:** Jeniffer Munyiva Mutua, John Mwaniki Njeru, Abednego Moki Musyoki

**Affiliations:** 1 Department of Laboratory Medicine, Kenyatta National Hospital, 20723-00202, Nairobi, Kenya; 2 Centre for Microbiology Research, Kenya Medical Research Institute, 19464-00200, Nairobi, Kenya; 3 Department of Medical Laboratory Sciences, Kenyatta University, 43844-00100, Nairobi, Kenya

**Keywords:** COVID-19, SARS-CoV-2, Multidrug-resistance, ESBL-resistance, Gram negative bacteria, ESBL-GNB

## Abstract

**Background:**

Bacterial infections in COVID-19 patients, especially those caused by multidrug-resistant gram-negative strains, are associated with increased morbidity, hospital stay and mortality. However, there is limited data on the epidemiology of extended-spectrum β-lactamase (ESBL)-producing bacteria in COVID-19 patients. Here, we assessed the prevalence and the factors associated with ESBL-producing gram-negative bacterial (GNB) infections among severely ill COVID-19 patients admitted in Kenyatta National Hospital (KNH), Kenya.

**Methods:**

We adopted a descriptive cross-sectional study design for patients admitted between October 2021 and February 2022, purposively recruiting 120 SARS-CoV- 2 infected participants based on clinical presentation. Demographics and clinical characteristics data were collected using structured questionnaires and case report forms. Clinical samples were collected and analyzed by standard microbiological methods in the KNH Microbiology laboratory and the Centre for Microbiology Research, Kenya Medical Research Institute.

**Results:**

GNB infections prevalence was 40.8%, majorly caused by ESBL—producers (67.3%) predominated by *Klebsiella pneumoniae* (45.5%). Generally, 73% of the ESBL producers harboured our target ESBL genes, mainly CTX-M-type (59%, 17/29) in *K. pneumoniae* (76.9%, 20/26)*.* GNB harbouring TEM-type (83%, 10/12) and SHV-type (100%, 7/7) genes showed ESBLs phenotypes and inhibitor resistance, mainly involving clavulanate, but most of them remained susceptible to tazobactam (60%, 6/10). SHV-type genes carrying ESBL producers showed resistance to both cefotaxime (CTX) and ceftazidime (CAZ) (*K. pneumoniae*), CAZ (*E. coli*) or CTX (*E. cloacae complex* and *K. pneumoniae*). About 87% (20/23) of isolates encoding CTX-M-type β-lactamases displayed CTX/ceftriaxone (CRO) resistance phenotype. About 42% of isolates with CTX-M-type β-lactamases only hydrolyzed ceftazidime (CAZ)*.* Isolates with OXA-type β-lactamases were resistant to CTX, CAZ, CRO, cefepime and aztreonam. Patients with comorbidities were 10 times more likely to have an ESBL-producing GNB infection (aOR = 9.86, 95%CI 1.30 – 74.63, p = 0.003).

**Conclusion:**

We report a high prevalence of ESBL-GNB infections in severely ill COVID-19 patients, predominantly due to *Klebsiella pneumoniae* harbouring CTX-M type ESBL genes. The patient’s underlying comorbidities increased the risk of ESBL-producing GNB infection. In COVID-19 pandemic, enhanced systematic and continuous surveillance of ESBL-producing GNB, strict adherence to infection control measures and antimicrobial stewardship policies are warranted in the current study setting.

## Background

Infection with Severe Acute Respiratory Syndrome Coronavirus-2 (SARS-CoV-2), the cause of Coronavirus Disease-2019 (COVID-19), suppresses host immunity through aberrant immune system activation and inflammatory cytokines overproduction [1]. Coupled with viral-induced epithelial damage [2], immune suppression favours bacterial colonization and subsequent infection [3]. Bacterial co-infections in COVID-19 patients, especially those caused by multidrug-resistant (MDR) Gram-negative strains, can result in prolonged hospitalization and higher mortality [4–6].Factors such as prior hospitalization, underlying medical conditions, immunosuppression, exposure to invasive medical procedures, and admission to intensive care units are associated with increased risk of MDR infections in non-COVID-19 patients [7].

Due to the lack of treatment guidelines at the beginning of the COVID-19 pandemic, most patients received broad-spectrum antibiotics [8]. Even though the impact of increased antibiotic use during the pandemic is still unclear, there was increased geographical distribution of carbapenemases, plasmid-encoded bacterial enzymes that hydrolyse carbapenem [8–10] in Latin America and the Caribbean [8]. However, the impact on the epidemiology of extended-spectrum β-lactamases (ESBL), with a similar transmission mechanism to carbapenemases, is unclear.

ESBLs are a group of bacterial enzymes that hydrolyse expanded spectrum β-lactam, thus mediating resistance against penicillins and cephalosporins [11]. These enzymes, produced predominantly by GNB, are worrisome because they can spread rapidly among clinical isolates through mobile genetic elements, which frequently co-harbour other non- β-lactam resistance genes, such as colistin [12, 13] aminoglycosides [14], and quinolones [15]. Surge in ESBL- producing bacterial infections can increase the use of carbapenems, which are among the drugs of last-resort in treatment of multidrug-resistant bacterial infections, posing a serious negative implication in clinical practice.

ESBL-production phenotype is mediated by several ESBL families, such as TEM, SHV, CTX-M, GES, PER, VEB, and BEL [11], with CTX-M-type β-lactamases mostly predominating [11]. Typically, beta-lactam combined with inhibitors, such as clavulanic acid, tazobactam or sulbactam, neutralize ESBL activities. Some TEM and SHV variants are resistant to inhibitors and, similar to other ESBLs, show geographical variation based on human mobility [11, 16, 17]. However, data on co-infections with ESBL-producing bacteria among COVID-19 patients in many developing countries, particularly in Sub-Saharan Africa, is limited. Therefore, we assessed the prevalence and risk factors for co-infection with ESBL-producing GNB among severely ill patients admitted in a Kenyan hospital unit dedicated to COVID-19 patients.

## Methods

### Study area, study design and data collection

We conducted this study in the Infectious Disease Unit (IDU), a ward dedicated to COVID-19 patients at Kenyatta National Hospital (KNH), Kenya, between October 2021 and February 2022. We adopted a descriptive cross-sectional study design among severely ill patients with confirmed (real-time reverse transcription and quantitative polymerase chain reaction (RT-qPCR) SARS-CoV-2 infection. Selection of severely ill COVID-19 participants was based on the WHO definition of severe COVID-19 illness; defined as, a critical condition where patients exhibit significant oxygen saturation deficits, impaired oxygen exchange in the lungs, rapid and labored breathing, or extensive lung infiltrates, all of which point to a severe respiratory and medical challenge associated with COVID-19 [18, 19]. This study purposively recruited 120 SARS-CoV-2 infected participants based on patients' clinical presentation suggesting bacteria infection as judged by the treating clinicians, and excluded those who, through their close relatives or legally authorized representative, declined consent to participate.

Data on demographics and clinical characteristics were collected using structured questionnaires and case report forms. Blood samples were collected directly into sterile blood culture bottles (bioMérieux, Marcy l´Etoile, France), observing the standard microbiological operating procedures [20]. Nasopharyngeal (NP) and oropharyngeal (OP) swabs (Sigma-Aldrich, India) and tracheal aspirate samples were collected by a licensed personnel into sterile containers, and transported in an ice box to the hospital Microbiology laboratory for immediate analysis.

### Bacteria isolation and identification

Bacterial isolation followed the standard microbiological methods [21]. We cultured NP/OP swabs and tracheal aspirate samples on sheep blood agar (Oxoid, United Kingdom) and MacConkey (Oxoid, United Kingdom), with an overnight incubation at 37 °C. Blood culture bottles were incubated in BACT/ALERT® VIRTUO 3D Microbial Detection Systems (bioMérieux, Marcy l'Etoile, France), followed by sub-culture for the positive samples onto chocolate blood agar (CBA) (Oxoid, United Kingdom), sheep blood agar (Oxoid, United Kingdom) and MacConkey (Oxoid, United Kingdom). After subculture, we incubated the plates in ambient air; and 5–10% CO_2_ at 37 °C overnight, followed by isolates' identification using VITEK Matrix-assisted laser desorption ionization–time-of-flight mass spectrometry (MALDI-TOF MS) (bioMérieux, Marcy l'Etoile, France). For quality control, we used *Escherichia coli* ATCC 8739. All the GNB isolates were transported to the Centre for Microbiology Research, Kenya Medical Research Institute (CMR-KEMRI) laboratories for further analysis.

### Screening for ESBL production

We screened the isolates for ESBL production using the Double Disk Synergy Test (DDST) [22]. A 0.5 McFarland-equivalent suspension of bacterial isolate  were plated on Mueller-Hinton Agar (MHA) and allowed to air dry for 3 min. Antibiotic disks, including cefotaxime (30 µg), ceftazidime (30 µg), and amoxicillin/clavulanic acid (20 µg/10 µg), were added at a 30 mm radius to radius distance and incubated overnight in ambient air at 37 ℃. An inhibition zone around the cefotaxime and/or ceftazidime that increased towards the β-lactam inhibitor was considered an ESBL producer. We used *K. pneumoniae* ATCC 700603 and *E. coli* ATCC 25922 for quality control.

ESBL production was also confirmed by the Phenotypic Confirmatory Disc Diffusion Test (PCDDT) [22]. Briefly, 0.5 McFarland of bacterium suspension was inoculated on MHA plate (Oxoid, United Kingdom) and allowed to air dry for 3 min. Antibiotics disks, including cefotaxime (30 µg), ceftazidime (30 µg), cefotaxime/clavulanic acid (30 µg/10 µg), and ceftazidime/clavulanic acid (30 µg/10 µg), were placed on the inoculated MHA plate, at a 30 mm (mm) radius to radius distance. The plates were incubated overnight in ambient air at 37 ℃. ESBL production was confirmed by observing an isolate with a > 5 mm-clear zone formed between the third-generation cephalosporin and the β-lactam inhibitor. *Klebsiella pneumoniae* ATCC 700603 and *Escherichia coli* ATCC 25922 were the control strains.

### Detection of ESBL resistance genes

ESBL producers were PCR-screened for SHV-, TEM-, OXA-1, and CTX-M- type ESBL genes using primers in Table 1. We extracted bacterial DNA using the heat lysis method [20] and followed the PCR protocol described by Kiiru et al*.* [22]. Briefly, 2 μl of DNA was added to 22 μl of PCR master mix (Bio-Rad Laboratories, Hercules, USA) with the target ESBL gene primers and loaded to a thermal cycler (Bio-Rad Laboratories, Hercules, USA), programmed as follows: denaturation at 95 °C for 6 min, annealing at 55 °C for 2 min, and final extension at 72 °C for 10 min. The amplification products were separated by gel electrophoresis (1.5% agar rose gel), stained with SYBR green dye and captured images by the Bio-Rad imaging system (Bio-Rad Laboratories, Hercules, USA). *Klebsiella pneumoniae* ATCC 700603 and *Escherichia coli* ATCC 25922 were the quality control organisms.

**Table 1:** Primer combinations used for detection of ESBL-encoding genes

Target Gene	Primer Name	Primer Sequence	Annealing Temp(°C)	Band Size (bp)	Source
TEM-type	TEM-F	5′-atgagtattcaacatttc cg-3′	50	867	[23]
	TEM-R	5′-ccaatgcttaatcagtga cg-3′			
SHV-type	SHV-F	5′-ttcgcctgtgtattatctccctg-3′	50	854	[24]
	SHV-R	5′-ttagcgttgccagtgytcg-3′			
OXA-1	OXA-F	5'-atgaaaaacacaatacatatcaacttc gc-3′	62	280	[23]
	OXA-R	5′-gtgtgtttagaatggtgatcgcat t-3′			
CTX-M-type	CTX-M-F	5′-atgtgcagyaacagtaarrgtkatg gc-3′	60	593	[23]
	CTX-M-R	5′-tgggtraartargtsaacagaaycagc gg-3′			

### Antimicrobial susceptibility testing

Further, we characterized the ESBL producers based on antimicrobial susceptibility to meropenem (MEM), colistin (COL), gentamicin (GEN), amikacin (AMK), aztreonam (ATM), ceftazidime (CAZ), cefotaxime (CTX), ceftriaxone (CRO), cefepime (FEP), piperacillin/tazobactam (TZP), amoxicillin/clavulanic acid (AMC), ampicillin/sulbactam (SAM), ciprofloxacin (CIP), and trimethoprim/sulfamethoxazole (SXT). We used VITEK 2 COMPACT system (bioMérieux, Marcy l'Etoile, France*)* to determine the isolates' antimicrobial susceptibility profile using Card AST GN 83, except for colistin and antibiogram interpreted based on CLSI (2021) guidelines [21]. Quality control organisms, *Pseudomonas aeruginosa* (ATCC 27853) and *Escherichia coli* (ATCC 25922), were used.

Colistin susceptibility testing was done by the Simple Disk diffusion method [25]. Using *Escherichia coli* ATCC 25922 and *P. aeruginosa* ATCC 27853 as quality control strains, we placed a 10 mg colistin disk on a 0.5 McFarland-equivalent bacterium suspension plated on modified Mueller–Hinton agar 30% (5.1 g/L) (Oxoid, United Kingdom), followed by an overnight incubation at 35 ℃ in 5% CO_2_. Minimum Inhibitory Concentrations (MICs), determined by broth microdilution following CLSI guidelines [21], were used to interpret the resultant inhibition zones. We defined multidrug-resistant organisms (MDRs) by resistance to three or more antibiotic classes [26].

### Data analysis and presentation

Statistical analysis was two-sided using STATA version 16. After describing continuous data in means and medians and categorical data in frequencies and percentages, we presented the data in tables and figures. Crude odds ratio (cOR) was analyzed using binary logic regression, with variables giving p-values ≤ 0.2 further computed by multiple regression analysis for adjusted odd ratio (aOR). The level of statistical significance was set at p-value ≤ 0.05, with a 95% Confidence Interval (CI), and statistically significant associations indicated in bold (Table 4).

## Results

### Demographic and clinical characteristics of patients with GNB infections

In this study, 49 (40.8%) of 120 severely ill COVID-19 patients had GNB infections. Of these, the majority were: adults aged above 60 years (36.7%), males (53.1%), married (73.5%), not vaccinated against COVID-19 (77.6%), comorbid (77.6%), and discharged (77.6%) after hospitalization for 6–10 days (51%), Table 2.

**Table 2:** Demographic and clinical characteristics of patients with GNB infections

Factors	Frequency, n = 49	Percent (%)
Age (years)		
Median (IQR)	49 (32–65)	
≤24	5	10.2
25–44	12	24.5
45–59	14	28.6
≥ 60	18	36.7
Gender		
Male	26	53.1
Female	23	46.9
Marital status		
Single	13	26.5
Married	36	73.5
Presence of comorbidity		
Yes	38	77.6
No	11	22.4
SARS-CoV-2 Vaccination status		
Yes	11	22.4
No	38	77.6
Hospitalization outcome		
Discharged	38	77.6
Died	11	22.4
Length of hospital stay (Days)		
Median (IQR)	9(5–12)	
Short stay (0–5)	11	22.4
Medium stay (6–10)	25	51.0
Long stay (> 10)	13	26.5

*IQR* interquartile range, *SARS-CoV-2* Severe acute respiratory syndrome coronavirus 2

### Spectrum of ESBL-producing GNB isolates in COVID-19 patients admitted in KNH-IDU

**Fig. 1:**
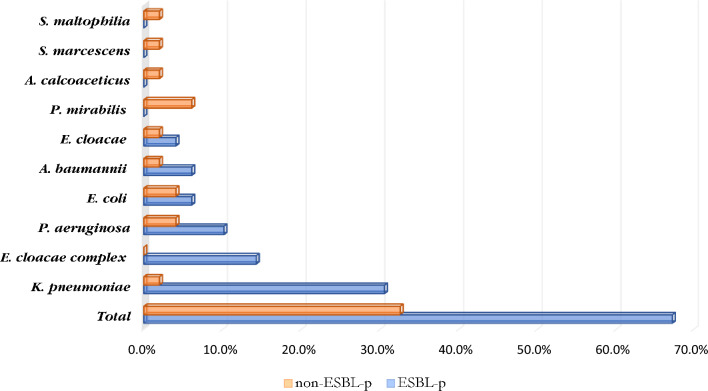
Spectrum of ESBL-producing GNB isolates in COVID-19 patients admitted in KNH-IDU. *ESBL-p* extended-spectrum beta-lactamases-producing, *non-ESBL-p* non-extended-spectrum beta-lactamases-producing

In this study, 49 out of 120 patients had GNB infections (40.8%). Of these, 33 (67.3%) were caused by ESBL – producers, predominated by *Klebsiella pneumoniae* (45.5%), *Enterobacter cloacae* complex (21.2%), *Acinetobacter baumannii* (12.1%), *Escherichia coli* (9.1%), *Pseudomonas aeruginosa* (6.1%) and *Enterobacter cloacae* (6.1%) Fig. 1**.** All *Enterobacter cloacae* complex (100%, 7/7) isolates were ESBL producers, whilst the other GNB isolates, including *Proteus mirabilis*, *Acinetobacter calcoaceticus, Serratia marcescens *and *Stenotrophomonas maltophilia*, were all non-ESBL producers Fig. 1.

### AMR genes carriage in ESBL-producing GNB isolates from COVID-19 patients admitted in KNH-IDU

**Fig. 2:**
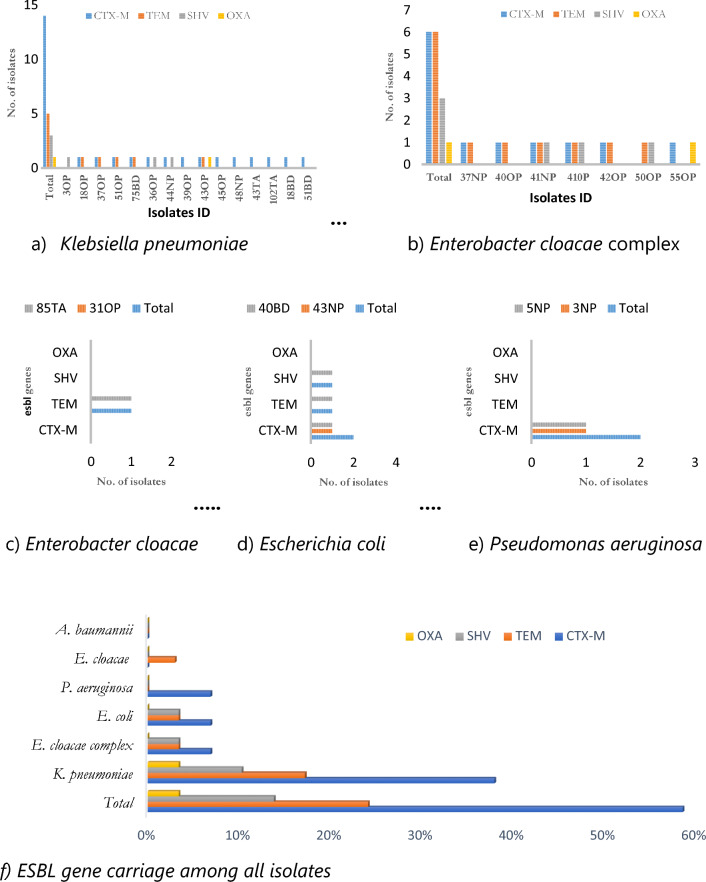
Distribution AMR genes among ESBL-producing GNB isolates from COVID-19 patients admitted in KNH-IDU. **a** *Klebsiella pneumoniae.* **b** *Enterobacter cloacae complex*. **c** *Enterobacter cloacae*. **d** *Escherichia coli*. **e** *Pseudomonas aeruginosa*. **f** ESBL gene carriage among all isolates. *TEM* TEM-type β-lactamase genes, *CTX-M* CTX-M-type β-lactamase genes, *SHV* SHV-type β-lactamase genes, *OXA-1* Oxacillinases -type β-lactamases 1, *ESBL* Extended Spectrum Beta Lactamase

We determined the occurrence of the most common ESBL genes, including CTX-M-type, TEM-type, SHV-type, and OXA-1, among the ESBL-producing bacterial isolates. All these genes were present in *Klebsiella pneumoniae*, predominated by CTX-M-type (60.9%, 14/23), and except for isolate 3OP harboring SHV gene only, other isolates encoded CTX-M-type genes, Fig. 2a. In *Enterobacter cloacae *complex, CTX-M-type and TEM were the principal ESBL genes (75%, 12/16), and similar to *K. pneumoniae,* AMR genes were present in all the ESBL-producing isolates, with OXA-1 genes as the minority, Fig. 2b. Fifty per cent (1/2) of ESBL-producing- *Enterobacter cloacae* isolates harbored a single ESBL gene, TEM, (Fig. 2c). About 67% (2/3) of ESBL-producing *E. coli *isolates encoded AMR genes targeted in this study, except OXA-type (Fig. 2d), whilst 40% (2/5) of ESBL producing- *Pseudomonas aeruginosa* isolates carried a single ESBL gene, CTX-M-type, Fig. 2e.

In general, 73% (24/33) of the ESBL-producing bacteria carried our study’s target AMR genes, mostly CTX-M-type (59%, 17/29), Fig. 2f. *Klebsiella pneumoniae* isolates harbored the majority of the study ESBL genes identified (76.9%, 20/26), and none of our target ESBL gene was detected in ESBL-producing *Acinetobacter baumannii*, Fig. 2f.

### Distribution of resistance genes among MDR phenotypes of ESBL-producing GNB

Isolates resistant to three or more antibiotic classes were considered multidrug-resistant organisms (MDRs) [19]. Eighty-three per cent of GNB harbouring TEM-type (10/12) and SHV-type (100%, 7/7) β-lactamases showed ESBL phenotypes and inhibitor resistance, mainly involving clavulanate but most of them remained susceptible to tazobactam (60%, 6/10), Table 3. One of TEM-type β-lactamases (8.3%, 1/12) from *E. cloacae complex* isolate (410P/NP) seemed to efficiently hydrolyze aztreonam (ATM) than cefotaxime (CTX) or ceftazidime (CAZ) Table 3.

**Table 3:** Distribution of resistance genes among MDR phenotypes of ESBL-producing GNB

Bacterial type	Isolate ID	MDR phenotype	ESBL genotype
*Klebsiella pneumoniae*	3OP	AMC/SAM/CTX/CAZ/CRO/ATM/CIP/COL	SHV
	43TA	SAM/CTX/CRO/ATM/SXT	CTX-M
	39OP	CTX/CRO/ATM/GEN/SXT/COL	CTX-M
	51BD	SAM/CTX/CAZ/CRO/ATM/GEN/SXT	CTX-M
	45OP	SAM/CTX/CRO/ATM/GEN/CIP/SXT/COL	CTX-M
	18BD	SAM/CTX/CAZ/CRO/FEP/ATM/GEN/SXT	CTX-M
	102TA	AMC/SAM/CTX/CAZ/CRO/ATM/GEN/CIP/SXT	CTX-M
	48NP	SAM/CTX/CAZ/CRO/ATM/GEN/CIP/SXT/COL	CTX-M
	36OP	SAM/CTX/CRO/ATM/GEN/SXT	CTX-M + SHV
	44NP	SAM/CTX/CRO/ATM/GEN/CIP/SXT	CTX-M + SHV
	51OP	CTX/CRO/ATM/GEN/SXT	CTX-M + TEM
	18OP	AMC/SAM/CTX/CRO/ATM/GEN/SXT	CTX-M + TEM
	37OP	SAM/CTX/CRO/ATM/GEN/CIP/SXT/COL	CTX-M + TEM
	75BD	AMC/SAM/CTX/CAZ/CRO/FEP/ATM/GEN/SXT	CTX-M + TEM
	43OP	CTX/CRO/ATM/GEN/SXT	CTX-M + TEM, OXA
*E. cloacae complex*	35OP	AMC/CTX/CRO/ATM/GEN/SXT	CTX-M + SHV
	37NP	AMC/CTX/CRO/ATM/GEN/SXT	CTX-M + TEM
	40OP	AMC/CTX/CRO/ATM/GEN/CIP/SXT	CTX-M + TEM
	420P	AMC/CTX/CAZ/CRO/ATM/GEN/SXT	CTX-M + TEM
	500P	AMC/CTX/CRO/ATM/GEN/SXT	TEM + SHV
	410P/NP	AMC/TZP/CRO/ATM/GEN/SXT	CTX-M + TEM + SHV
	550P	AMC/TZP/CTX/CAZ/CRO/FEP/ATM/MEM/GEN/CIP/SXT	CTX-M + OXA
*Enterobacter cloacae*	85TA	AMC/CTX/CAZ/CRO/ATM	TEM
	31OP	AMC/CTX/CAZ/CRO/ATM/GEN/SXT	*ND*
*Pseudomonas aeruginosa*	3NP	CTX/CRO/ATM/GEN/SXT	CTX-M
	5NP	TZP/CTX/CAZ/FEP	CTX-M
*Escherichia coli*	43NP	SAM/CTX/CRO/GEN/CIP/SXT/COL	CTX-M
	40BD	AMC/SAM/TZP/CAZ/CRO/FEP/GEN/CIP/SXT	CTX-M + TEM + SHV
	40NP	SAM/CTX/CRO/GEN/CIP/SXT	*ND*
*Acinetobacter baumannii*	21TA	TZP/CTX/CAZ/CRO/FEP/MEM/CIP/SXT	*ND*
	36BD	SAM/TZP/CTX/CAZ/CRO/FEP/GEN/CIP/SXT	*ND*
	94TA	TZP/CTX/CAZ/CRO/FEP/GEN/CIP/SXT	*ND*
	112TA	CTX/CRO/FEP/CIP/SXT	*ND*

*AMC* amoxicillin/clavulanate, *SAM* ampicillin/sulbactam, *TZP* piperacillin/tazobactam, *CTX* cefotaxime, *CAZ* ceftazidime, *CRO* ceftriaxone, *FEP* cefepime, *ATM* aztreonam, *MEM* meropenem, *COL* colistin, *GEN* gentamicin; *CIP* ciprofloxacin, *SXT* trimethoprim/sulfamethoxazole, *ESBLs extended spectrum β- lactamases, TEM* temoneira -type β-lactamases, *CTX-M* Cefotaxime-hydrolysing b-lactamase isolated in Munich-type β-lactamases, *SHV* sulfhydryl variable-type β-lactamases, *OXA-1* Oxacillinases -type β-lactamases 1, *ND* Not Detected

In this study, SHV-type genes carrying ESBL producers showed resistance to both CTX and CAZ (*K. pneumoniae* isolate, 3OP), CAZ (*E. coli*, 40BD) or CTX (*E. cloacae* complex (50OP) and *K. pneumoniae* (36OP and 44 NP). About 13% (3/24) of bacterial isolates encoding CTX-M-type β-lactamases did not display the typical cefotaxime/ceftriaxone (CTX/CRO) resistance phenotype of the early CTX-M variants. About 42% (5/12) of ESBL-producing GNB with CTX-M-type β-lactamases only hydrolyzed ceftazidime (CAZ), Table 3.

The OXA-1 β-lactamases detected were resistant to third (cefotaxime, CTX; ceftazidime, CAZ; ceftriaxone, CRO) fourth-generation cephalosporins (cefepime, FEP) and monobactam (aztreonam, ATM). One of OXA-type b-lactamases isolated from *E. cloacae* complex isolates (55OP) showed carbapenemases activity, Table 3.

### Factors associated with ESBL-producing GNB infections among COVID-19 patients admitted in KNH-IDU

Multivariable analysis established that severely ill COVID-19 patients with comorbidities were about ten (10) times more likely to have an infection caused by ESBL-producing GNB (aOR = 9.86, 95%CI 1.30 – 74.63, p = 0.003). Male gender was also a risk factor for infection with ESBL-producing GNB, although there was no independent association (cOR = 9.97(2.32–42.85), p = 0.002), Table 4.

**Table 4:** Factors associated with ESBL-producing GNB infections among COVID-19 patients admitted in KNH-IDU

Factors	ESBL producer		cOR(95%CI)	P-value	aOR(95%CI)	P-value
	Yes n (%)	No n (%)				
Age (years)						
≤24	2 (6.1)	3 (18.8)	3.90 (0.49–30.76)	0.196	13.95 (0.74–23.11)	0.078
25–44	9 (27.3)	3 (18.8)	0.87 (0.16–4.58)	0.866	2.02 (0.21–19.44)	0.542
45–59	9 (27.3)	5 (31.3)	1.44 (0.32–6.49)	0.632	3.67 (0.40–33.96)	0.252
≥60	13 (39.4)	5 (31.3)	Ref.		Ref	
Gender						
Male	23 (69.7)	3 (18.8)	9.97 (2.32–42.85)	**0.002**	3.38 (0.59–19.43)	0.172
Female	10 (30.3)	13 (81.3)	Ref.		Ref.	
Marital status						
Single	7 (21.2)	6 (37.5)	0.45 (0.12–1.67)	0.231		
Married	26 (78.8)	10 (62.5)	Ref			
Presence of comorbidity						
Yes	30 (90.9)	8 (50.0)	10.0 (2.15–46.61)	**0.003**	9.86 (1.30–74.63)	**0.027**
No	3 (9.1)	8 (50)			Ref.	
SARS-CoV-2 Vaccination status						
Yes	8 (24.2)	3 (18.8)	1.39 (0.31–6.13)	0.483		
No	25 (75.8)	13 (81.3)	Ref.			
Hospitalization outcome						
Alive	27 (81.8)	11 (68.8)	2.05 (0.52–8.12)	0.466		
Died	6 (18.2)	5 (31.3)	Ref.			
Length of hospital stay (Days)						
Short stay (0–5)	9 (27.3)	2 (12.5)	Ref.		Ref.	
Medium stay (6–10)	17 (51.5)	8 (50)	0.26 (0.04–1.70)	0.159	0.39 (0.04–4.02)	0.425
Long stay (> 10)	7 (21.2)	6 (37.5)	0.55 (0.14–2.18)	0.393	0.43 (0.06–2.96)	0.39

Statistically significant associations are indicated in bold

*cOR* crude odds ratio, *aOR* adjusted odds ratio, *ESBLs* extended spectrum beta-lactamases, *Ref* Reference, *SARS-CoV-2* severe acute respiratory syndrome coronavirus 2, *CI* confidence interval,

## Discussion

In this study, we screened 49 gram negative bacterial (GNB) isolates for ESBL production. Of these, 67.3% were ESBL – producers, predominated by *K. pneumoniae* (30.6%). Contrary to our findings, Lemenand and colleagues reported a decreasing proportion of ESBL among *E. coli* infections (2.9%) during the COVID-19 pandemic in France [27]. The study by Lemenand et al. focused only on single bacteria, *E. coli*, and their data might not be generalizable to countries differently impacted by the COVID-19 pandemic. Karataş et al. observed a significant decrease in ESBL-P Enterobacterales during the pandemic period compared to the pre-pandemic era [28]. In the study by Karatas and others, not all participants were COVID-19 patients. Different from Karatas et al., we targeted all GNB in severely ill COVID-19 patients confirmed by real-time reverse transcription and quantitative polymerase chain reaction (RT-qPCR), and admitted in critical care unit. This could possibly explain the high prevalence of ESBL producer isolates in our study. In the current study, the prevalence of ESBL-producing GNB infections among COVID-19 patients was higher than that reported in non-COVID-19 patients in East African (42%) and Kenya (47%) [29]. In a recent study among Kenyan children at the point of hospital discharge, the prevalence of ESBL-producing *E. coli* was 44.3% [30]. Together, these reports suggest a higher prevalence of ESBL-producing GNB in severely ill COVID-19 patients admitted in ICU in our setting.

*Klebsiella pneumoniae* (30.6%) was the predominant ESBL producer among GNB isolates from severely ill COVID-19 patients. Even though data on ESBL-producing bacteria in COVID-19 patients is limited, in the general population, *E. coli* and *K. pneumoniae* [31–34] seems to be the most common ESBL producers. Our finding may infer similarity in ESBL-producing bacteria profiles among COVID-19 and non-COVID patients. In the hospital environment, GNB can acquire and transfer ESBL genes via mobile genetic elements such as plasmids and transposons, and predominating ESBL producers may vary geographically depending on environmental sanitary status, adherence to infection prevention and control protocols and antimicrobial stewardship policies [35, 36].

To decipher the antimicrobial resistance (AMR), AMR gene carriage among ESBL phenotypes, the commonly reported ESBL genes in bacteria, (CTX-M- type, TEM, SHV and OXA-1) were detected by PCR. About 73% of the ESBL-producing GNB encoded our target AMR genes, mostly CTX-M-type (59%, 17/29), with *K. pneumoniae* harbouring the majority (76.9%) of the genes. Emeraud et al. documented a nosocomial outbreak of ESBL producing *K. pneumoniae* carrying CTX-M-15 in a French intensive care unit dedicated to COVID-19 during the first wave of the pandemic [37], however, information on ESBL gene carriage among ESBL producing GNB that cause infections in COVID-19 patients is limited. Before the year 2000, SHV- and TEM–type enzymes were the most predominant ESBLs worldwide [38] but have since been outnumbered by CTX-M ESBLs in non-COVID19 patients [39, 40, 11, 34]. Therefore, our findings suggest a similar ESBL gene carriage among bacterial isolates from COVID-19 patients and the general population. Clinically, the CTX-M-producing bacterial infections are treated using carbapenems, thus promoting the spread of potentially untreatable carbapenemase-producing bacterial infections [38].

In this work, 83% of TEM-type and SHV-type (100%) β-lactamases showed ESBLs phenotypes and inhibitor resistance, mainly involving clavulanate, but mostly remained susceptible to tazobactam (60%, 6/10). Inhibitor-resistant variants emerge following mutations that result in one, two or three amino acid substitutions in the parental enzymes [40]. These mutations confer resistance to clavulanate and sulbactam but not tazobactam and avibactam [11, 41–43]. TEM-type β-lactamases (8.3%) from *E. cloacae* complex isolate, 410P/NP, seemed to efficiently hydrolyze aztreonam (ATM) than cefotaxime (CTX) or ceftazidime (CAZ), a phenotype that was reported in TEM-184 with Q6K, E104K, I127V, R164S and M182T amino acid substitutions [44, 45]. SHV-type ESBL producers showed resistance to both CTX and CAZ (*K. pneumoniae* isolate, 3OP), CAZ (*E. coli*, 40BD) or CTX (*E. cloacae* complex (50OP) and *K. pneumoniae* (36OP and 44 NP).

SHV-type enzymes mutations that result in the substitution of lysine (Lys238) with serine (Ser) and lysine (Lys240) with glutamic acid (Glu) play a critical role in the efficient hydrolysis of ceftazidime and cefotaxime, respectively [11]. About 87% (3/24) of isolates harboring CTX-M-type β-lactamases in our study displayed the typical cefotaxime/ceftriaxone (CTX/CRO) resistance phenotype observed with the early CTX-M variants [11], and about 42% of the isolates showed ceftazidime (CAZ) resistant phenotypes. The CAZ- resistance has been reported in CTX-M-15 [46, 47] and CTX-M-27 variants [48].

In our study, the OXA-1 β-lactamases detected were resistant to third (cefotaxime, CTX; ceftazidime, CAZ; ceftriaxone, CRO) and fourth (cefepime, FEP)-generation cephalosporins and monobactam (aztreonam, ATM). One of OXA-1 b-lactamases isolated from *E. cloacae* complex isolates (55OP) had carbapenemase activity. These enzymes are known to have hydrolytic activity against penicillins and cephalosporins, including third-and/or fourth-generations [49]. OXA-1/OXA-30 [50, 51] and OXA-31 [52] variants is associated with FEP resistance. OXA-48 derivative, OXA-163 and OXA-405, OXA-58, OXA-143, and OXA-235 [52] have carbapenemase activity. OXA-48 β-lactamases mediating carbapenem resistance among ESBL-producing *Escherichia coli* and *Klebsiella pneumoniae* isolates were describe in a Turkish university hospital [53]. Though we did not elucidate molecular variants of the ESBL genes, these published reports suggest the possible inhibitor-resistance mechanisms among bacterial isolates in our study.

Severely ill COVID-19 patients with comorbidities were at higher risk of infection by ESBL-producing bacteria. Greco and others found that COVID-19 patients with comorbidities, such as diabetes mellitus and hypertension, were at increased risk of co-infections in Italy [54]. In a multi-centre study by He and others on clinical characteristics of COVID-19 patients with clinically diagnosed bacterial co-infection, patients with cardiovascular comorbidities were more likely to have clinically diagnosed bacterial co-infection [55]. In the current study, the most common comorbidities were cancer (17%), kidney disease (16%), diabetes (14.9%), hypertension (11.7%), haematological disorders (7.4%) and HIV/AIDS (6.4%).

This study has some limitations. As a single centre study, the data obtained may not be generalizable to other hospitals within our locality and therefore, a larger study is recommended to determine this epidemiology against the general patient population. Additionally, the purposive sampling may have subjected it to selection bias, and due to resource constrains, we were unable to elucidate the molecular variants of the ESBL genes detected. However, this study highlights the need for systematic and continuous surveillance of multidrug-resistant bacteria among SARS-CoV-2 infected persons in the hospital to inform AMR prevention interventions in line with national and global action plans.

## Conclusion

We report a high prevalence of ESBL-GNB infections in severely ill COVID-19 patients, predominantly due to *Klebsiella pneumoniae* harbouring CTX-M type ESBL genes. The patient’s underlying comorbidities increased the risk of ESBL-producing GNB infection. In COVID-19 pandemic, enhanced systematic and continuous surveillance of ESBL-producing GNB, strict adherence to infection control measures and antimicrobial stewardship policies are warranted in the current study setting.

## Data Availability

The datasets used and/or analysed during the current study are available from the corresponding author on reasonable request.

## Abbreviations

AMR: Antimicrobial Resistance
CTX-M: Cefotaxime-hydrolysing β-lactamase isolated in Munich
ESBL: Extended Spectrum β-Lactamases
MDR: Multidrug Resistant
OXA: Oxacillinases
SARS-CoV-2: Severe Acute Respiratory Syndrome-Corona Virus 2
SHV: Sulfhydryl reagent variable
TEM: Temoneira*, the patient infected with the first isolate expressing TEM-1*
